# Optimization of Salt-Leaching Parameters for Gelatin/Na_2_Ti_3_O_7_ Scaffolds Using a Mixture Design Experiment

**DOI:** 10.3390/polym14030559

**Published:** 2022-01-29

**Authors:** Rittichai Sangkatip, Wipoo Sriseubsai, Kunlanan Kiatkittipong, Kaona Jongwuttanaruk

**Affiliations:** 1 Department of Industrial Engineering, School of Engineering, King Mongkut’s Institute of Technology Ladkrabang, Bangkok 10520, Thailand; 58601034@kmitl.ac.th; 2 Department of Chemical Engineering, School of Engineering, King Mongkut’s Institute of Technology Ladkrabang, Bangkok 10520, Thailand; kunlanan.kia@kmitl.ac.th; 3 Department of Industrial Engineering, Faculty of Engineering, Rajamangala University of Technology Thanyaburi, Chiang Mai 12110, Thailand; kaona.j@en.rmutt.ac.th

**Keywords:** gelatin, titanium dioxide, mixture design

## Abstract

The purpose of this research was to learn the formation of biomedical scaffold material from gelatin by using titanate (Na_2_Ti_3_O_7_), which is a newly synthesized derivative of titanium dioxide (TiO_2_) with gelatin. It was prepared by mixed several solutions and cross-linked molecules by heating and salt-leaching. The biomedical scaffold was formed, and its porosity depended on the size of the salt crystal. The mixture was designed by using a mixture design with three factors: gelatin, titanate, and deionized water to determine the optimal mixture for the tensile strength of the biomedical scaffold. The microstructure of the biomedical scaffold was studied using scanning electron microscopy (SEM). The findings revealed that Na_2_Ti_3_O_7_ thoroughly pen-extracted the biomedical scaffold, and the tensile strength of the gelatin/titanate scaffold was higher than the biomedical scaffold, which was formed using pure gelatin. By using the mixture design technique, the 14.73% gelatin, 0.2% Na_2_Ti_3_O_7_, and 85.07% DI water got the highest yield of tensile strength (1508.15 kP). This was an about 4.88% increase in the tensile strength property when compared with using TiO_2_.

## 1. Introduction

Tissue engineering is a new and exciting technique which has the potential to create tissues and organs de novo. It involves the in vitro seeding and attachment of human cells onto a scaffold. These cells then proliferate, migrate, and differentiate into specific tissue while secreting the extracellular matrix components required to create the tissue. It is evident, therefore, that the choice of scaffold is crucial to enable cells to behave in the required manner to produce tissues and organs of the desired shape and size. Current scaffolds, made by conventional scaffold fabrication techniques, are generally foams of synthetic polymers. The cells do not necessarily recognize such surfaces, and most importantly, cells cannot migrate more than 500 µm from the surface. The lack of oxygen and nutrient supply governs this depth. Solid freeform fabrication (SFF) uses layer-manufacturing strategies to create physical objects directly from computer-generated models. It can improve current scaffold design by controlling scaffold parameters such as pore size, porosity, and pore distribution, as well as incorporating an artificial vascular system, thereby increasing the mass transport of oxygen and nutrients into the interior of the scaffold and supporting cellular growth in that region [1,2,3]. The process of tissue formation is based on three major principles, which include material engineering, cell biology, and biochemical engineering. Tissue formation starts from the development of biomaterials (i.e., materials well suited to work in human tissues) that serve as a biomedical scaffold; natural biomaterials such as collagen and gelatin are the most popular biomaterials [4]. Biomedical scaffolds are used to culture isolated cells, regulate cells to achieve sufficient numbers, and induce change in tissues during differentiation. Recently, research on the development of medical materials from natural biomaterials, such as collagen, gelatin, chitosan bacteria, cellulose, seaweed extract, etc., has gained popularity. Mechanical properties of materials are important for developing reliable and effective medical materials [5]. Titanium dioxide (TiO_2_) is one of the most popular materials used to make medical materials; for example, titanium dioxide mixed with chitosan collagen can be used to make biomedical scaffolds for wound repair [4,6]. Titanium dioxide is suitable for making biomedical scaffolds, has mechanical properties that are well suited to the body, and is highly porous, nontoxic to cells, and protects against *Staphylococcus aureus* infection [7,8,9].

Therefore, we were interested in studying the use of titanate, a derivative synthesized from titanium dioxide (TiO_2_) as a filler material [10,11]. We wanted to determine the suitability of the material to form a cell scaffold after being mixed with gelatin. We aimed to use this mixture to increase the tensile strength of various polymers [12,13,14].

It is important to design a matrix with mechanical properties (stress and strain) that mimic the properties of tissue in the immediate surrounding area of the defect. In bone tissue engineering, for instance, an over-designed matrix around the implant site can actuate bone resorption, while an under-designed matrix may fail as a mechanical support to the framework. The mechanical properties can be varied through the proper selection of material, critical development of composite structures, and the general porosity of the framework [15].

## 2. Materials and Methods

### 2.1. Materials

Powdered gelatin from porcine skin, 180 G Bloom, Type B from Fluke Analytical, and 2,2,2-trifluoroethanol (TFE) (purity 99.0%) from Sigma-Aldrich, Bangkok, Thailand were used. Commercial titanium dioxide (TiO_2_) was used as the raw material to synthesize titanate (Na_2_Ti_3_O_7_), which was used for the experiment. Titanate was synthesized through alkaline hydrothermal reaction with 0.5 g of titanium dioxide (TiO_2_) as a precursor and 20 mL of sodium hydroxide (NaOH) at the concentration of 10 molar. The titanium dioxide mixture and sodium hydroxide were blended for 90 min, then put in cylinder pressure, which was a Teflon-lined autoclave, and put into the oven at 200 °C for 24 h. Finally, they were allowed to cool to room temperature. Then, the substance was washed with deionized water until the acidity and alkalinity (pH) was approximately 7, and then it was baked at a temperature of 80 °C for 24 h [11]. The synthesized titanate had a specific surface area (BET) of 10 m^2^/g and an average primary particle size of 197 nm; 100% of the titanate was in the form of anatase.

### 2.2. Mixture Design

This study was based on a constrained mixture design principle or vertex model. The experimental design had three parameters, which consisted of gelatin, Na_2_Ti_3_O_7_, and DI water. We used the Minitab 17 software for statistical analyses. We customized the software to display three factors denoted as A, B, and C. A: Gelatin: Gelatin and collagen were used to produce the scaffold for fibroblast cell culture. The properties of scaffolds obtained from type A and type B gelatin were compared to the scaffold obtained from collagen, which is widely used as a skin substitute. B: Na_2_Ti_3_O_7_: Titanate Ribbon (Na_2_Ti_3_O_7_) was synthesized to study the mechanical properties and C: DI water. The ratio in which the three substances were mixed is presented in Table 1.

#### 2.2.1. Sample Preparation

Based on the principles of mixture design, the three raw materials (gelatin, titanate, and DI water) were stirred for 30 min at 50 °C at a definite ratio, followed by the addition of sodium chloride. The mixture was stirred well and poured into a mold to cool down to room temperature. Then, the mixture was washed with deionized water to remove NaCl, dried, and subjected to dehydrothermal treatment (DHT), where it was baked at 140 °C for 48 h.

#### 2.2.2. Mechanical Characterization

Tensile strength is the main mechanical property of scaffolds made from Na_2_Ti_3_O_7_ with gelatin, which is discussed. The methods to measure mechanical properties are also presented in this brief review article. Finally, some perspectives are given for the future development of Na_2_Ti_3_O_7_ in tissue engineering. Tensile tests were performed using a universal tensile machine (Zwick Z010) at room temperature, according to the range of tests described by ASTM D3574. The speed of the crosshead was maintained at 10 mm/min. The tests were repeated three times, and the average was considered for further analysis.

## 3. Results and Discussion

### 3.1. Experimental Design and Results

Based on the conditions mentioned above, the 27 experimental trials were performed with three replicates per test condition. The results of the tensile tests are shown in Table 2.

### 3.2. Residual Plots for Response

**Figure 1:**
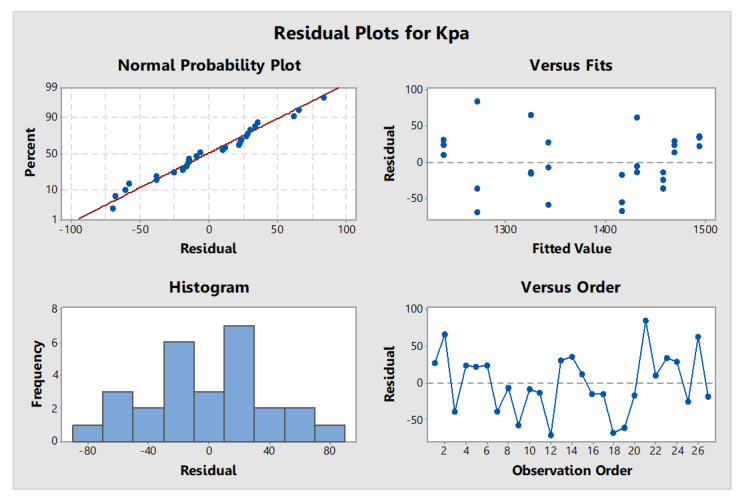
Residual plots for response average (kPa).

The obtained results were used to determine the quality of the data by model verification analysis. If the data were distributed normally, they were used for model verification; otherwise, they were used to analyze the coefficient of determination (R-squared) and variance (ANOVA). The quality of the data was tested using the (1) normal distribution test, (2) data independence test, and (3) test of variance stability (Figure 1).

### 3.3. Analysis of Variance (ANOVA)

Analysis of variance was performed to test the tensile strength [16]. Results were considered to be statistically significant for *p* < 0.05 (Table 3), and it was found that the interaction term between the amount of gelatin and the amount of titanate (Na_2_Ti_3_O_7_) was statistically significant (*p* = 0.220). The amount of gelatin and the amount of DI water were not significantly related (*p* > 0.05). The coefficient of determination (R^2^) shows the percentage variation in the dependent variables that is explained by the independent variables in a regression analysis. The regression analysis for the response surface methodology shown in Table 3 indicated that the coefficient of determination was high (R^2^ = 92.23%), which indicated that the independent variables (the amounts of gelatin, titanate (Na_2_Ti_3_O_7_), and DI water) could explain the variation in the independent variables (the amounts of gelatin, titanate (Na_2_Ti_3_O_7_), and DI water) by 90.47%. Hence, the model was used to form a prediction equation to determine an accurate and appropriate response value.

### 3.4. Results of the Response Surface Methodology

**Figure 2:**
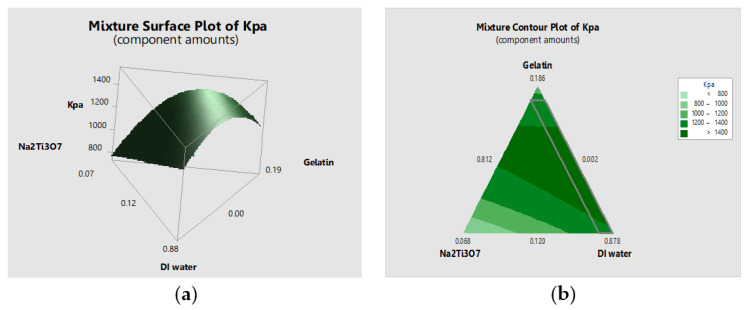
(**a**) Mixture surface and (**b**) mixture contour plot.

The mixture surface and the mixture contour plot were determined from the mixture regression equation of the response surface for tensile strength. Figure 2 shows that the response surface for tensile strength in the biomedical-scaffold-forming process among gelatin, titanate (Na_2_Ti_3_O_7_), and DI water increased with the increase in the amounts of gelatin and Na_2_Ti_3_O_7_.

### 3.5. Analysis of the Experiment

**Figure 3:**
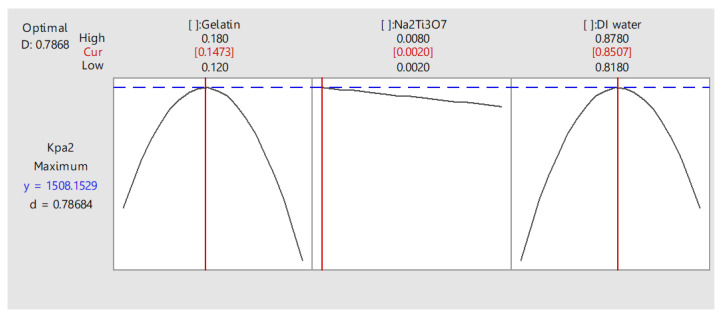
Multiple response prediction.

A response optimizer function to determine the most appropriate value of the factors was used to efficiently obtain the tensile strength. We also used a function to find the most appropriate parameter of the factors and measure composite desirability (D). Composite desirability ranged from 0 to 1. When D was equal to 1, the result was favorable for the overall response. The response optimizer function was used to determine the most appropriate value of the factors. The results showed that 14.73% gelatin, 0.2% titanate (Na_2_Ti_3_O_7_), and 85.07% DI water, or gelatin/Na_2_Ti_3_O_7_ in the ratio of 80/20 yielded the highest tensile strength of 1508.15 kPa, and desirability at 0.786, as shown in Figure 3.

Gelatin and Na_2_Ti_3_O_7_ consisting of ratios of 100/0, 90/10, 80/20, 70/30, and 60/40 have been studied using the degradation test and swelling test and by analyzing the surface morphology and pore size.

### 3.6. Degradation of Gelatin/Na_2_Ti_3_O_7_ Biomedical Scaffolds

Lysozyme from chicken egg white (Bio Basic Inc., Markham ON, Canada), at a concentration of 31.2 u/mL (0.1 mg/mL PBS buffer), was added to the biomedical scaffolds for degradation. Then, the mixture was incubated in an oven at 37 °C for 54 h. The sample was incubated in an enzyme-free buffer. After incubation, the sample was thoroughly rinsed with purified water to remove the enzymes, dried, and weighed multiple times, i.e., after 0.5, 1, 1.5, 24, 48, and 54 h. We calculated the degree of enzymatic degradation [16,17] from the following equation:

(1) $$Weight\;remain\;\left(\%\right)=100-\left[\frac{\left(W_{0}-W_{f}\right)}{W_{0}}\;\times \;100\right]$$

where:

$W_{0}$ is the initial weight of the biomedical scaffold;

$W_{f}$ is the final weight of the biomedical scaffold.

**Figure 4:**
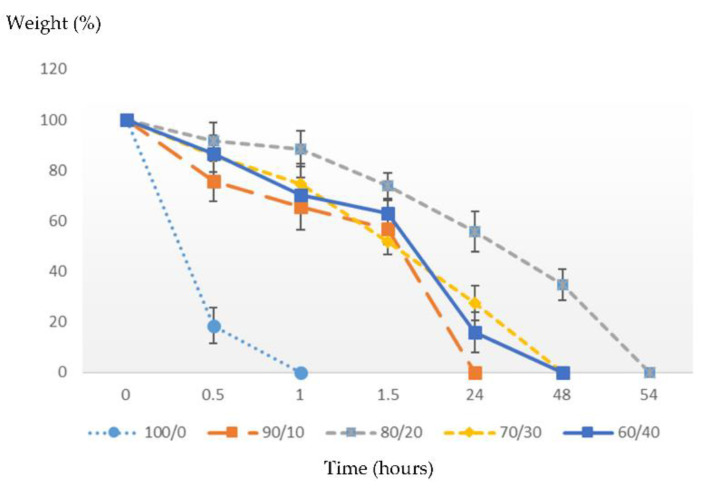
Result of the degradation of gelatin/Na_2_Ti_3_O_7_ biomedical scaffolds. (n = 20).

The pure gelatin biomedical scaffolds and gelatin/Na_2_Ti_3_O_7_ biomedical scaffolds were compared based on the analysis of the degradation method [18,19], and it was found that the biomedical scaffolds made of pure gelatin (ratio of gelatin/Na_2_Ti_3_O_7_ is 100/0) were degraded in one hour, but the biomedical scaffolds made of the gelatin/Na_2_Ti_3_O_7_ mixture (90/10 ratio) required 24 h for complete degradation. Moreover, mixtures of gelatin/Na_2_Ti_3_O_7_ at 60/40 and 70/30 degraded in 48 h, and the mixture of gelatin/Na_2_Ti_3_O_7_ ratio at 80/20 degraded in 54 h, as shown in Figure 4.

### 3.7. Swelling Test

To evaluate the percentage difference in the dry weight and wet weight of the biomedical scaffold, the swelling test was performed. The dried biomedical scaffold was weighed and soaked in a PBS buffer solution at a pH of 7.4 and 37 °C for three hours [20,21]. Then, both sides of the scaffold were wiped with low lint paper for 10 s per side and weighed immediately. The dry weight and wet weight were then used to calculate the swelling ratio using the following formula:

(2) $$\mathrm{Swelling}\;\mathrm{ratio}\;=\frac{W_{so}-W_{o}}{W_{0}}$$

where:

$W_{so}$ is the weight of the scaffold after its water content is absorbed;

$W_{0}$ is the initial weight of the biomedical scaffold.

**Figure 5:**
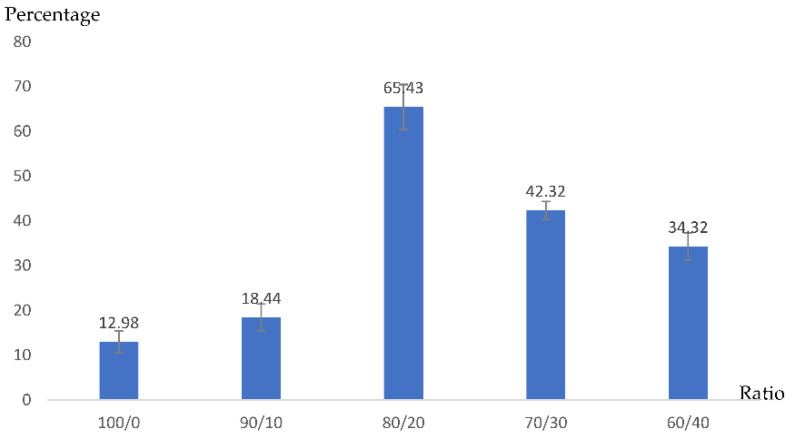
Swelling ratio of gelatin/Na_2_Ti_3_O_7_ scaffold via salt-leaching technique. (n = 20).

Figure 5 shows that the biomedical scaffolds obtained using the salt-leaching technique with a mixture ratio of 100/0 had a swelling ratio of 12.98%. The highest swelling ratio of 65.43% was obtained from the salt-leaching technique for the gelatin and Na_2_Ti_3_O_7_ mixture, mixed at a ratio of 80/20.

### 3.8. Surface Morphology of Biomedical Scaffolds

**Figure 6:**
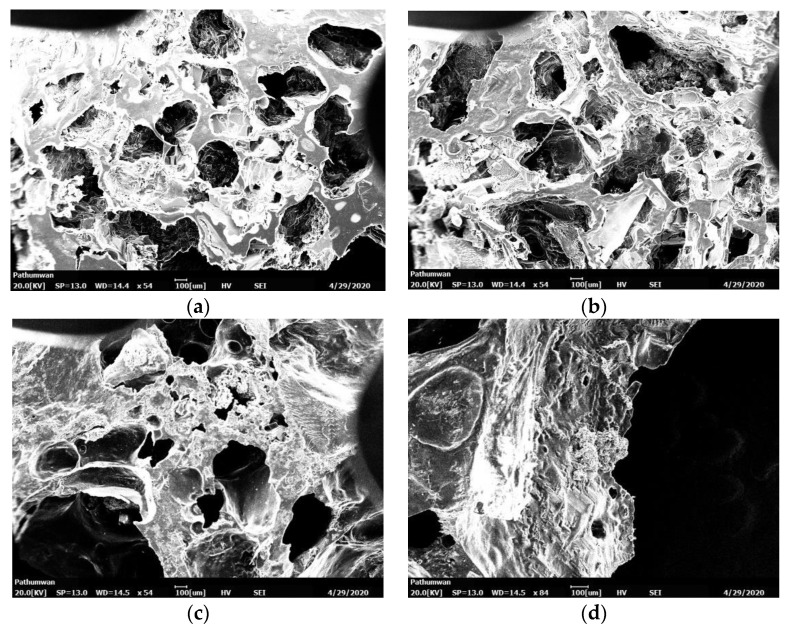
SEM images of the scaffold obtained via the salt-leaching technique with gelatin/Na_2_Ti_3_O_7_ mixed in a ratio of (**a**) 90/10, (**b**) 80/20, (**c**) 70/30, and (**d**) 60/40; all biomedical scaffolds underwent dehydrothermal treatment for 48 h.

The morphology of the biomedical scaffold was analyzed via scanning electron microscopy (SEM, JSM-5610LV, JEOL) at a voltage of 20 kV after coating the surface of the sample with gold. The pore size was measured at least 20 times, and the average diameter was calculated as shown in Table 4. The images of the pores of the biomedical scaffolds were revealed after they underwent dehydrothermal treatment (DHT) at 140 °C for 48 h as shown in Figure 6. The diameter of the pores of the gelatin/Na_2_Ti_3_O_7_ changed as well. When a greater amount of Na_2_Ti_3_O_7_ was added to the mixture, the pores sized was reduced. Figure 6d shows that the biomedical scaffold does not have any pores. Figure 6c shows highly variable pore sizes, and Figure 6a,b show that the diameter of the pores of the gelatin/Na_2_Ti_3_O_7_ biomedical scaffolds ranged from 409.6 to 580.4 µm.

## 4. Conclusions

The formation of a cell scaffold was studied by mixing gelatin with titanate (Na_2_Ti_3_O_7_), a derivative synthesized from titanium dioxide (TiO_2_). The solutions were mixed, and the molecules were cross-linked using heating and salt-leaching methods to obtain porous cell biomedical scaffolds. The porosity of the scaffolds was related to the size of the salt crystals. A mixture design was used for the experiment with three factors, which included gelatin, titanate, and deionized water. Based on the conditions mentioned above, mixtures were prepared for 27 experimental trials (runs) with three replicates. Model verification demonstrated that the model was appropriate, with data showing a normal distribution, independence, and variance stability. The variance analysis of the amount of gelatin, titanate (Na_2_Ti_3_O_7_), and DI water showed that the interaction term between the amount of gelatin and titanate (Na_2_Ti_3_O_7_) was significant (*p* = 0.220). However, the amount of gelatin and DI water were not significantly related. The regression analysis for the response surface methodology indicated that the coefficient of determination was high (0.9223), which implied that the independent variables (the amounts of gelatin, titanate (Na_2_Ti_3_O_7_), and DI water) explained the variation in the dependent variables (the amounts of gelatin, titanate (Na_2_Ti_3_O_7_), and DI water) by 90.47%. Thus, the model could be used to make a prediction equation to find an accurate and appropriate response value. To find the most appropriate value of the factors to efficiently determine the tensile strength, we used a response optimizer function. We also used a function to find the most appropriate parameter of the factors and measure composite desirability (D). Composite desirability ranged from 0 to 1. When D was equal to 1, the results were favorable for the overall response. The response optimizer function, which was used to determine the most appropriate value of the factors, showed that 14.73% gelatin, 0.2% titanate (Na_2_Ti_3_O_7_), and 85.07% DI water, or gelatin/Na_2_Ti_3_O_7_ in a ratio of 80/20 yielded the highest tensile strength (1508.15 kPa). Regarding the surface morphology of biomedical scaffolds gelatin/Na_2_Ti_3_O_7_ biomedical scaffolds, the distribution of portion of matter was found on the structure of gelatin. In comparison, the porosity of the gelatin/Na_2_Ti_3_O_7_ scaffold at an 80/20 ratio was 440.3 µm (on average), with a tensile strength of 1508.15 kPa and 60/40 ratio; pores could not be seen, and it could not be used as a biomedical scaffold. The difference in the porosity of these two substances affected the pore size and the tensile strength. Our study revealed that the biomedical scaffolds derived from the salt-leaching technique with pure gelatin (mixture ratio of 100/0) had a swelling ratio of 12.98%. The highest swelling ratio was 65.43%. Additionally, it was found that the gelatin/Na_2_Ti_3_O_7_ scaffolds, mixed at a ratio of 80/20, degraded in 54 h.

Titanate (Na_2_Ti_3_O_7_) was mixed with gelatin to improve the mechanical properties of the cell scaffold. There were some literatures showed that the increasing of nano-TiO_2_ content in gelatin and chitosan mixture improved the tensile strength of the scaffold gradually up to 1438 kPa [22]. When compared with this study, it is shown that adding Na_2_Ti_3_O_7_ and gelatin to a biomedical scaffold in the ratio of gelatin 80% and Na_2_Ti_3_O_7_ 20% could improve the mechanical tensile strength to 1508.15 kPa, or an increase of 4.88%. The biomedical scaffold had large pores and was mechanically stronger; therefore, it can be applied for tissue engineering via the salt-leaching technique. Thus, the gelatin/Na_2_Ti_3_O_7_ scaffold (salt-leaching using powder) technique may be a promising method for fabricating a scaffold in lab-scale experiments, because it is easy to use and facile to fabricate a well-interconnected scaffold with well-defined geometry. Composite scaffolds could be promising candidates for use in wound-healing dressings. Finally, an optimization analysis was carried out to select the best conditions for the process.

## Figures and Tables

**Table 1:** List of the raw materials used to synthesize the gelatin/Na_2_Ti_3_O_7_ scaffold.

Factors	Factor Levels	
	Low Level	High Level
A: Gelatin (g)	0	20
B: Na_2_Ti_3_O_7_ (g)	0	2
C: DI water (ml)	0	87.8

**Table 2:** Experimental design and results.

Run Order	Factors			Tensile Test (KPa.)
	A: Gelatin	B: Na_2_Ti_3_O_7_	C: DI Water	
1	0.120	0.002	0.878	1370.372
2	0.120	0.008	0.872	1391.098
3	0.180	0.002	0.818	1233.898
4	0.180	0.008	0.812	1262.019
5	0.150	0.005	0.845	1515.289
6	0.135	0.004	0.862	1493.043
7	0.135	0.007	0.859	1419.788
8	0.165	0.004	0.832	1425.541
9	0.165	0.007	0.829	1359.31
10	0.120	0.002	0.878	1334.372
11	0.120	0.008	0.872	1311.098
12	0.180	0.002	0.818	1201.898
13	0.180	0.008	0.812	1268.506
14	0.150	0.005	0.845	1529.289
15	0.135	0.004	0.862	1481.043
16	0.135	0.007	0.859	1443.324
17	0.165	0.004	0.832	1416.839
18	0.165	0.007	0.829	1348.72
19	0.120	0.002	0.878	1282.372
20	0.120	0.008	0.872	1309.098
21	0.180	0.002	0.818	1355.898
22	0.180	0.008	0.812	1248.506
23	0.150	0.005	0.845	1527.289
24	0.135	0.004	0.862	1497.043
25	0.135	0.007	0.859	1432.963
26	0.165	0.004	0.832	1493.541
27	0.165	0.007	0.829	1397.634

**Table 3:** Summary of the analysis of variance (ANOVA).

Source	DF	Seq SS	Adj MS	F	*p*
Regression	4	200,642	200,642	25.45	0.001
Linear	2	25,681	177,327	44.98	0.001
Quadratic	2	174,961	174,961	44.38	0.003
Gelatin * Na_2_Ti_3_O_7_	1	201	3141	1.59	0.220
Gelatin * DI water	1	174,759	174,759	88.67	0.005
Residual Error	22	43,362	1971		
Lack-of-Fit	4	16,271	4068	2.70	0.063
Pure Error	18	27,091	1505		
Total	26	244,004			

R-Sq = 92.23% R-Sq (pred) = 90.47% R-Sq (adj) = 99.00%. * is physical mixing

**Table 4:** The pore size of the scaffolds produced via the salt-leaching technique.

Gelatin/Na_2_Ti_3_O_7_	Range of Diameter (µm)	Average Diameter (µm) ± SD
90/10	498.3–580.4	532.2 ± 54.3
80/20	409.6–528.9	440.3 ± 64.2
70/30	576.4–1207.4	704.34 ± 97.3
60/40	0	0

## Data Availability

The data presented in this study are available in this article.
